# Effect of Different Postharvest Pre-Cooling Treatments on Quality of Water Bamboo Shoots (*Zizania latifolia*) during Refrigerated Storage

**DOI:** 10.3390/plants13202856

**Published:** 2024-10-12

**Authors:** Shuwen Tang, Zhongyi Xu, Chenwei Chen, Jing Xie

**Affiliations:** 1 College of Food Science and Technology, Shanghai Ocean University, Shanghai 201306, China; 2 National Experimental Teaching Demonstration Center for Food Science and Engineering, Shanghai Ocean University, Shanghai 201306, China; 3 Shanghai Professional Technology Service Platform on Cold Chain Equipment Performance and Energy Saving Evaluation, Shanghai 201306, China

**Keywords:** vegetables, pretreatment, preservation, quality

## Abstract

Post-harvest pre-cooling of water bamboo shoots (WBS) [*Zizania latifolia*] can effectively delay its quality deterioration. Six types of pre-cooling treatments were used to pre-cooling post-harvest WBS, including cold slightly acidic electrolytic water pre-cooling (CSAEW), cold water pre-cooling (CWPC), vacuum pre-cooling (VPC), strong wind pre-cooling (SWPC), refrigerator pre-cooling (RPC), and fluid ice pre-cooling (FIPC). The effects of different pre-cooling treatments on the quality of refrigerated WBS were investigated. The results showed that the FIPC treatment was harmful to the storage quality of WBS, while the other five pre-cooling treatments could extend the shelf life of WBS to some extent. These pre-cooling treatments can inhibit the respiration of WBS, slow down its weight loss and lignification process, and maintain its relatively high levels of nutrient content and antioxidant activity. The CSAEW treatment outperformed other treatments in terms of bactericidal action and microbiological content control for WBS during storage. The protective effect of CSAEW treatment on the storage quality of WBS was relatively the best, and extended the shelf life of WBS by 12 days compared to the control group. This study indicated that the CSAEW pre-cooling treatment offers a new choice for pre-cooling root vegetables.

## 1. Introduction

Fresh vegetables constitute a cornerstone of a nutritious diet for humans, which is indispensable to daily life. During post-harvest phases, including transportation, storage, and marketing, vegetables are highly vulnerable to senescence and quality degradation, leading to the loss and wastage of up to 28% to 55% [1]. Various technologies have been employed to preserve vegetables, including low-temperature storage [2,3], modified atmosphere packaging [4,5,6], irradiation [7], preservative treatments [8], and combinations of these techniques [9,10]. These technologies primarily extend the shelf life of vegetables by modulating ambient conditions, reducing respiration and water loss, or suppressing surface microbial populations. However, the quality degradation induced by residual field heat post-harvest was usually neglected. Most vegetables are harvested at elevated temperatures and retain significant field heat. This not only accelerates respiration and transpiration of vegetables, but also fosters an environment conducive to microbial proliferation, severely compromising their quality.

Pre-cooling is a technology employed to rapidly reduce the temperature of vegetables immediately post-harvest, effectively eliminating residual field heat [11]. Pre-cooling can quickly slow down the metabolic and physiological processes of vegetables, keeping their quality before storage. Common pre-cooling technologies encompass cold storage pre-cooling, strong wind pre-cooling (SWPC), vacuum pre-cooling (VPC), chilled water pre-cooling, and ice water pre-cooling. Each pre-cooling technology has its advantages, disadvantages, and specific applications. Given the distinct characteristics of various vegetables, their optimal pre-cooling processes vary accordingly. Tao et al. [12] investigated the effect of pre-cooling methods of natural convection pre-cooling, strong wind pre-cooling (SWPC), vacuum pre-cooling (VPC), cold water pre-cooling (CWPC), electrolytic water pre-cooling, and fluid ice pre-cooling (FIPC) on bok choi (*Brassica rapa* var. *chinensis*), and demonstrated that VPC was the best pre-cooling method for it. Garrido et al. [13] investigated the effects of cold storage pre-cooling, SWPC, water cooling, and VPC on the quality of baby spinach. They found that the water pre-cooling treatment enhanced its sensory attributes. Patel et al. [14] examined hydrocooling (cold water was sprayed on the surface), SWPC, and chilled water dipping pre-cooling of tomatoes, demonstrating that chilled water dipping pre-cooling was the most effective method for tomatoes. Mittal et al. [15] assessed the impact of water pre-cooling, cold storage pre-cooling, SWPC, and VPC on the shelf-life of mushrooms, concluding that VPC was the optimal pre-cooling method for mushrooms. Defraeye et al. [16] showed that SWPC were suitable for citrus fruits. Zhang et al. [17] showed that cold water pre-cooling (CWPC) and refrigerator pre-cooling (RPC) were the most suitable methods for yellow peaches. Zhang et al. [18] found that SWPC and ice water pre-cooling were the most successful pre-cooling methods for sweet corn before storage.

Slightly acidic electrolyzed water (SAEW) is electrolyzed by hydrochloric acid water, with a pH range of 5.0 to 6.5 and an effective chlorine (HClO) concentration of 10–30 ppm. It is environmentally friendly and exhibits effective germicidal action, showing extensive application potential in the food industry [19,20]. Koide et al. [21] showed that SAEW exhibited a significant bactericidal effect on fresh-cut purple kale, proving to be as effective as or even superior to sodium hypochlorite solution. Li et al. [22] found that slightly acidic electrolyzed water was effective in preserving the quality of freshly cut eggplant. Zhang et al. [19] showed that SAEW enhanced the storage quality of carambola fruit by reducing its respiration rate and increasing its bioactivity and nutrient content. However, there was less research on the application of SAEW (low-temperature) for pre-cooling vegetables.

Water bamboo shoots (WBS) [*Zizania latifolia*], a perennial aquatic grass extensively cultivated in China and South Asia, belonging to the genus *Zizania*, is abundant in dietary fibers and essential nutrients. WBS is favored for its crisp texture, palatable taste, and nutritional value. However, the quality of post-harvest WBS is prone to deteriorate during storage and its shelf-life is about 2–3 days at room temperature. Few studies on the pre-cooling of WBS have been reported. The main causes of post-harvest senescence and rapid quality deterioration in WBS are physiological metabolism and lignification [23]. The main manifestation of its lignification is an increase in hardness. Therefore, reducing respiration swiftly and decelerating lignification after postharvest are critical for extending the shelf life of WBS. Utilizing low temperatures is a widely employed strategy to inhibit respiratory activity and reduce physiological and metabolic processes in vegetables. Several technologies have been shown to inhibit the synthesis of lignin-related enzymes and slow the lignification process in WBS, such as radio frequency [24], nitric oxide [25], high-pressure carbon monoxide [26], high hydrostatic pressure [27], UV-C irradiation [28], and melatonin [23]. However, few studies on the effect of pre-cooling treatments on the quality of WBS during storage have been reported, as well as the result of pre-cooling treatments on the lignification of WBS.

In this study, six pre-cooling treatments were used for pre-cooling the freshly harvested WBS, including cold slightly acidic electrolytic water pre-cooling (CSAEW), CWPC, VPC, SWPC, RPC, and FIPC. To identify the most suitable pre-cooling treatment for WBS, the effects of different pre-cooling treatments on the quality of WBS were investigated.

## 2. Materials and Methods

### 2.1. Materials and Pre-Cooling Treatments

**Figure 1:**
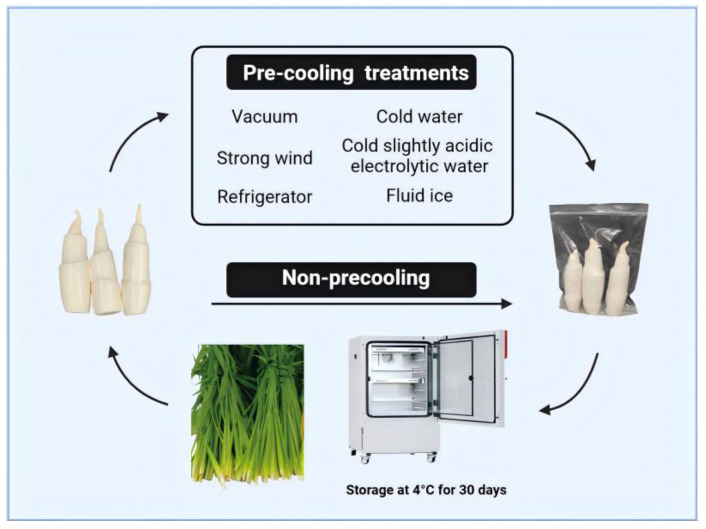
The schematic diagram of the pretreatment and packaging process for Water Bamboo Shoots (WBS) [*Zizania latifolia*].

The WBS were harvested from nearby farms (Shanghai, China) and transported back to the laboratory as soon as possible. The WBS’s hulls were peeled off. The WBS samples which were clean with similar size, similar color, and undamaged surfaces were selected. The schematic diagram of the pretreatment and packaging process for WBS is shown in Figure 1. The initial temperature of the WBS was measured before pre-cooling. Then, the WBS samples were pre-cooled by following different pre-cooling treatments. During the pre-cooling process, the center temperature of the sample and the ambient temperature were determined using T-type thermocouples. The pre-cooling process was completed until the center temperature of the WBS dropped to 4 °C. The dried WBS samples were put into polyethylene bags after the moisture on the surface of the WBS was wiped away. The packaged samples were stored in a refrigerator (KMF 240, Binder Environmental Test Equipment (Shanghai) Co., Ltd., Shanghai, China) at 4 °C. The WBS samples were taken out for measuring of their quality indicator every 6 days. The WBS without any pre-cooling treatment was set as the control group (CK). The six different pre-cooling treatments were as follows:

(1) CSAEW: the WBS samples were pre-cooled by placing them in slightly acidic electrolytic water at 1 °C. The slightly acidic electrolytic water was prepared through a PG3.0 water electrolysis device (Shanghai Ouai Environmental Protection Technology Co. Ltd, Shanghai, China) by using dilute hydrochloric acid (6%, *w*/*w*) and tap water. The parameters of slightly acidic electrolytic water were as follows: pH 6.32 ± 0.02, redox potential ORP value of 862.40 ± 12.25 mV, and effective chlorine content of 30.00 ± 1.26 mg/L.
(2) CWPC: the WBS samples were pre-cooled by placing them in cold water at 1 °C.
(3) VPC: the WBS samples were put into a vacuum pre-cooling machine (VAC-0.2 type, Shanghai Fresh Green Vacuum Preservation Equipment Co., Ltd., Shanghai, China) for pre-cooling. The surface of the WBS was sprayed with an appropriate amount of water. The final temperature was 4 °C and the final pressure was 750 Pa.
(4) SWPC: the WBS samples were pre-cooled by placing them in trays in a strong wind pre-cooling machine (DJ-12type, Zhengfa Technology Co., Ltd., Kunming, China) with a speed of 8 m/s at a temperature of 1 °C.
(5) RPC: the WBS samples were pre-cooled by placing them in a refrigerator with natural convection at 1 °C.
(6) FIPC: the WBS samples were pre-cooled in the prepared fluid ice. The fluid ice was prepared by passing brine with a concentration of 3.3% through a seawater fluidized ice maker (R-1000W-SP type, Jiangsu Nantong Ruiyou Industry and Trade Co., Ltd., Nantong, China) at a temperature of −1.8 ± 0.5 °C.

### 2.2. Determination of WBS Quality

#### 2.2.1. Respiration Rate

Referring to the method reported by Li et al. [29], the respiration rate of WBS during storage was determined by the alkali absorption method.

#### 2.2.2. Whiteness

The whiteness of WBS indicates the degree of its surface whiteness, which was determined according to the method reported by Wen [28]. A chroma meter (CR-400, Tokyo, Japan) was used to measure the color (L*, a*, and b*) of the WBS in the middle part. Each group was measured three times and the average value was taken. The whiteness was calculated as follows:

(1) $$\mathrm{Whiteness}=100-\sqrt{\left[{\left(100-L^{*}\right)}^{2}+a^{*}+b^{*}\right]}$$

#### 2.2.3. Weight Loss

The water on the surface of the WBS was dried and the WBS was weighed. Weight loss was determined using the following formula:

(2) $$\mathrm{Weight}\;\mathrm{loss}=\frac{M-m}{M}\times 100\%$$

M is the mass of WBS before storage, (g); m is the mass of WBS after storage, (g).

#### 2.2.4. Hardness

The hardness of WBS was determined using a GY-3 (Zhejiang AIRUP Co., Ltd., Hangzhou, China) durometer with a probe diameter of 0.8 cm. The middle part of the WBS was selected for testing. The unit was kg/cm^2^.

#### 2.2.5. Lignin Content

The lignin content was determined using a lignin content assay kit (BC4200, Beijing Solarbio Science & Technology Co., Ltd., Beijing, China) according to the manufacturer’s instructions. The phenolic hydroxyl groups in lignin were acetylated with characteristic absorption peaks at 280 nm, and their absorbance values were positively correlated with the lignin content. The sample was dried and acetylated, and the lignin content was calculated by measuring the absorbance value after completion of the determination.

#### 2.2.6. Phenylalanine Ammonialyase (PAL) Activity

The PAL activity was determined using the PAL activity assay kit (BC0210, Beijing Solarbio Science & Technology Co., Ltd., Beijing, China) according to the manufacturer’s instructions. PAL catalyzes the conversion of L-phenylalanine into trans-cinnamic acid and ammonia, with trans-cinnamic acid exhibiting maximum absorption at 290 nm. One unit of enzyme activity was defined as a 0.1 change in absorbance at 290 nm per gram of tissue, per milliliter of reaction mixture, per minute.

#### 2.2.7. Peroxidase (POD) Activity

The POD activity was determined using a POD activity assay kit (BC0090, Beijing Solarbio Science & Technology Co., Ltd., Beijing, China) according to the manufacturer’s instructions. POD catalyzes the oxidation of specific substrates by H_2_O_2_, with a characteristic light absorption at 470 nm. One unit of enzyme activity was defined as a 0.01 change in absorbance at 470 nm per gram of tissue, per milliliter of reaction system, per minute.

#### 2.2.8. Soluble Solids Content

Three samples were selected at random, and sliced in their middle. The samples were ground and squeezed to extract the juice. Then, it was filtered through multiple layers of gauze and the soluble solid content was determined using a hand-held refractometer (PR32a, ATAGO, Tokyo, Japan). The measurement was repeated three times for each group and the average value was taken.

#### 2.2.9. Soluble Sugar Content

The soluble sugar content was determined using a plant soluble sugar content assay kit (BC0035, Beijing Solarbio Science & Technology Co., Ltd., Beijing, China) according to the manufacturer’s instructions. Soluble sugar content was determined using anthrone colorimetry, with absorbance measured at 620 nm for the treated samples.

#### 2.2.10. Total Protein Content

The total protein content was determined using a total protein quantitative assay kit (A045-2-2, Nanjing Jiancheng Bioengineering Institute, Nanjing, China) according to the manufacturer’s instructions. Total protein content was determined using the Coomassie Brilliant Blue method, in which the -NH_3_^+^ group of the protein molecules binds with the anionic form of the reddish-brown Coomassie Brilliant Blue, causing the solution to turn blue. The protein content was calculated by measuring the absorbance at 595 nm.

#### 2.2.11. Ascorbic Acid Content

The ascorbic acid content was determined using an ascorbic acid/total ascorbic acid (AsA/T-AsA) assay kit (BC4630, Beijing Solarbio Science & Technology Co., Ltd., Beijing, China) according to the manufacturer’s instructions. Ascorbic acid possesses reducing properties, enabling it to reduce Fe^3+^ to Fe^2+^. The Fe^2+^ ions form a pink complex with 2,2′-bipyridine, which exhibits a characteristic absorption peak at 525 nm. This reaction can be used to determine the ascorbic acid content in the sample.

#### 2.2.12. Malondialdehyde (MDA) Content

The MDA content was determined using the MDA content assay kit (BC0020, Beijing Solarbio Science & Technology Co., Ltd., Beijing, China) according to the manufacturer’s instructions. MDA reacts with thiobarbituric acid under acidic conditions and high temperature to form a reddish-brown compound, trimethylchuan (3,5,5-trimethyloxazole-2,4-dione), which exhibits a maximum absorption at 532 nm and can be used to estimate MDA content.

#### 2.2.13. Total Bacterial Colony Count

Referring to the national standard GB4789.2-2022 [30], 5 g of WBS mixed with 45 mL of sterile saline was homogenized for 2 min. The diluted mixture was injected into the medium and it was incubated at 37 °C for 48 h. The total bacterial colony count was counted and the result was expressed as log_10_ CFU/g.

#### 2.2.14. Statistical Analysis

The experiment was repeated three times for each variable and the average was taken. All data were analyzed with the IBM SPSS Statistics 26 software (SPSS Inc., Chicago, IL, USA). The Duncan’s multiple range test was used to determine the significance of each mean property value (*p* < 0.05) after a one-way analysis of variance (ANOVA) was completed. Plotting was performed with Origin 2022 (9.900225).

## 3. Results and Discussion

### 3.1. Pre-Cooling Curve

**Figure 2:**
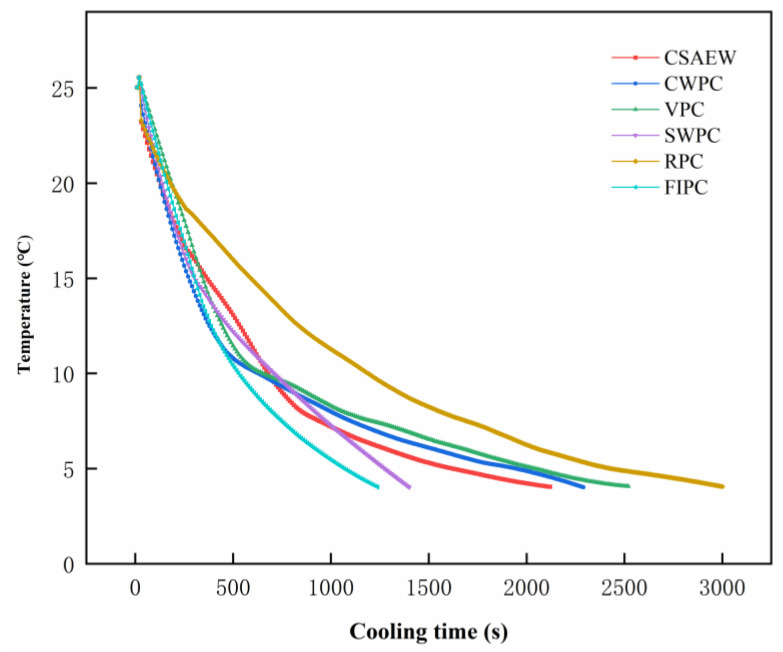
The time–temperature variation curves of WBS using different pre-cooling treatments. The different abbreviations CSAEW, CWPC, VPC, SWPC, RPC, FIPC in the figure represent the cold slightly acidic electrolytic water pre-cooling, cold water pre-cooling, vacuum pre-cooling, strong wind pre-cooling, refrigerator pre-cooling, and fluid ice pre-cooling, respectively.

Pre-cooling can delay the maturation and senescence of vegetables by eliminating field heat and respiratory heat accumulated post-harvest [31]. The post-harvest quality and shelf life of vegetables are significantly influenced by the pre-cooling rate [32], which is a critical factor in selecting the optimal pre-cooling technique. Figure 2 shows the time–temperature variation curves of WBS using different pre-cooling treatments. The pre-cooling process commenced at a uniform temperature of 25 °C and ceased when the central temperature of the WBS reached 4 °C. Differences in cooling rate were observed among the various pre-cooling methods, with the FIPC group cooling the fastest, followed by the SWPC, CSAEW, CWPC, VPC, and RPC groups in descending order. The FIPC group’s quick cooling could be due to the low temperature of fluid ice (−1.8 ± 0.5 °C) and effective heat transfer. Since water has a higher heat transfer coefficient than air, water cooling should theoretically be faster than air cooling [33]. However, the SWPC group had a pre-cooling speed second only to FIPC group. The reason may be that use of stronger wind speeds in a relatively small space speeds up the circulating flow of air and removes heat from the WBS more quickly, thus reaching the pre-cooling final temperature more quickly than water pre-cooling. The CSAEW and CWPC treatment groups had intermediate pre-cooling rates, but they did not cause moisture loss. The relatively slow pre-cooling speed of the VPC treatment group can be attributed to VPC, which relied on evaporative cooling. Considering the small specific surface area, the skin’s water retention capacity, and the tighter internal structure of WBS, evaporation was hindered, slowing the heat removal process. Zhu et al. [34] also concluded that root vegetables were unsuitable for VPC. They also found that the RPC treatment took the longest time, as it relied on natural air convection to dissipate heat from WBS, aligning with our experimental findings.

### 3.2. Respiration Rate

**Figure 3:**
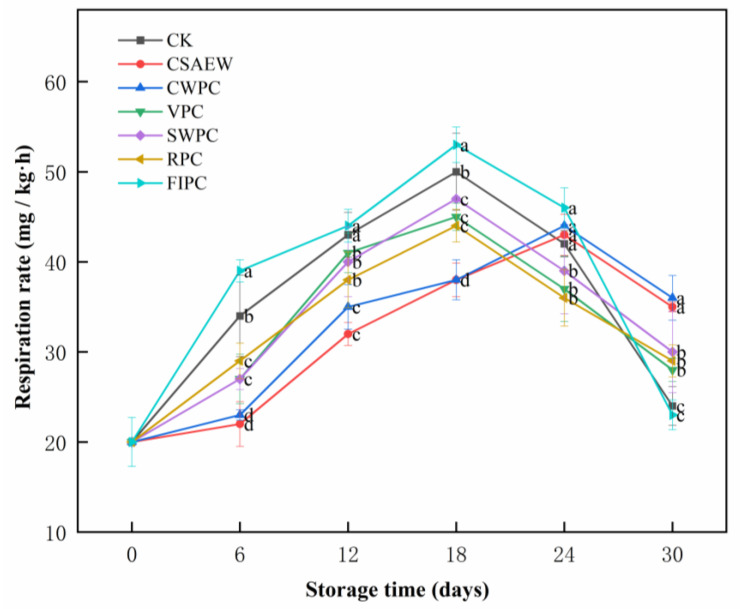
The change in respiration rate of WBS subjected to different pre-cooling treatments during storage. The different abbreviations CK, CSAEW, CWPC, VPC, SWPC, RPC, FIPC in the figure represent control group, cold slightly acidic electrolytic water pre-cooling, cold water pre-cooling, vacuum pre-cooling, strong wind pre-cooling, refrigerator pre-cooling, and fluid ice pre-cooling, respectively. Same superscript lowercase letters in a column indicate no significant differences (*p* > 0.05).

Respiration is the biochemical process through which plants convert organic matter into energy. The intensity of respiration increases with tissue injury or degradation, and it is also affected by microbial infection, making it a crucial predictor of fruit and vegetable senescence [19,35]. WBS are a respiratory-climacteric vegetable, with their ripening process marked by the occurrence of respiratory peaks. Figure 3 shows the change in respiration rate of WBS subjected to different pre-cooling treatments during storage. The respiration rate of WBS increased first and then progressively declined, indicating the onset of senescence in the later stage of storage. The respiration peak occurred on day 24 in the CSAEW and CWPC groups, and occurred on day 18 in the other groups. The respiration rate of WBS in the FIPC group was consistently higher than that in the CK group. This may be due to the low temperature of the FIPC treatment and the mechanical damage incurred during de-hulling, which reduced its resistance to cold and increased its respiration rate. Subzero temperatures can freeze water molecules in plants, leading to frostbite. Frostbite damages the cellular structure of plants, impairing their normal physiological functions [36]. The respiratory rate in the CSAEW and CWPC groups was not only low during storage (significantly different from other groups, *p* < 0.05) but also delayed the emergence of the respiration peak, indicating that these pre-cooling treatments effectively inhibit the respiration of WBS, maintaining low physiological activity and thus delaying aging. The CSAEW treatment exhibited a slightly better inhibitory effect on the respiratory respiration of WBS, likely because it can reduce the rate of infection by pathogenic microorganisms and enhance the disease resistance of WBS. Zhang et al. [19] demonstrated that SAEW reduced respiration in carambola fruit by inhibiting microbial growth. The VPC, SWPC, and RPC treatments all suppressed the respiration of WBS to some extent, but the CSAEW and CWPC groups were markedly more effective. The reason might be that WBS had a high water content (approximately 93%), which was more prone to loss in VPC and SWPC treatment. Han et al. [37] investigated the effects of pre-cooling on the post-harvest storage quality of black mulberry and found that pre-cooling significantly reduced respiratory intensity and delayed physiological activity during storage. Zhang et al. [17] demonstrated that CWPC delayed peak respiration in yellow peach and reduced its ethylene production, thus postponing its ripening and senescence.

### 3.3. Whiteness and Weight Loss

**Figure 4:**
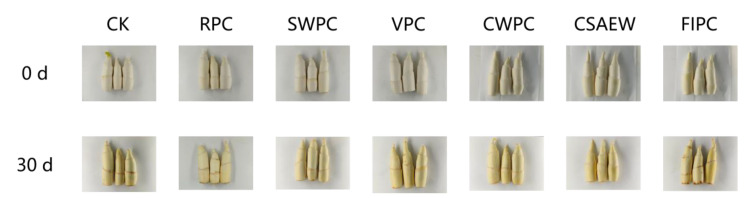
The photographs of the appearance of WBS subjected to different pre-cooling treatments during storage. The different abbreviations CK, RPC, SWPC, VPC, CWPC, CSAEW, FIPC in the figure represent control group, refrigerator pre-cooling, strong wind pre-cooling, vacuum pre-cooling, cold water pre-cooling, cold slightly acidic electrolytic water pre-cooling, and fluid ice pre-cooling, respectively. Same superscript lowercase letters in a column indicate no significant differences (*p* > 0.05).

**Figure 5:**
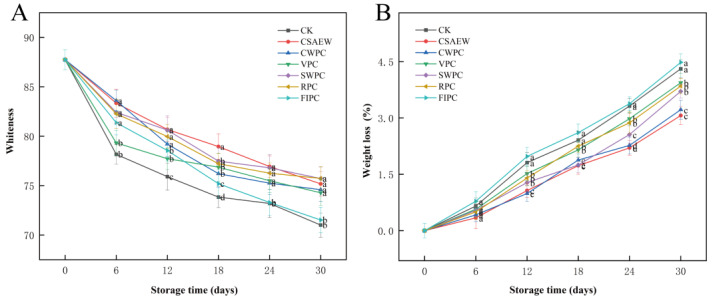
The change in (**A**) whiteness and (**B**) weight loss of WBS subjected to different pre-cooling treatments during storage. The different abbreviations CK, CSAEW, CWPC, VPC, SWPC, RPC, FIPC in the figure represent control group, cold slightly acidic electrolytic water pre-cooling, cold water pre-cooling, vacuum pre-cooling, s strong wind pre-cooling, refrigerator pre-cooling, and fluid ice pre-cooling, respectively. Same superscript lowercase letters in a column indicate no significant differences (*p* > 0.05).

To a certain extent, appearance indicates the quality of storage for vegetables. Figure 4 shows photographs of WBS subjected to different pre-cooling treatments during storage. After completing the pre-cooling, there was no significant change in the appearance of the groups. The appearance quality of WBS progressively declined in all treatment groups with increasing storage time. By the 30th day, the CSAEW and CWPC groups exhibited no significant browning, maintaining superior appearance quality compared to other treatment groups. The diminished appearance quality in the CK and FIPC groups was primarily due to extensive surface browning, resulting in poorer appearance. The RPC, SWPC, and VPC groups exhibited slight browning during storage, leading to moderate appearance quality. Color visually reflects the quality of vegetables to consumers. Figure 5A shows the change in whiteness of WBS subjected to different pre-cooling treatments during storage. It was evident that the whiteness in all groups gradually decreased with increased storage time. Compared to the CK and FIPC groups, the other five pre-cooling treatments were able to partially inhibit the reduction in whiteness of WBS (*p* < 0.05). This observation was consistent with the appearance in the photographs.

Weight loss is an important indicator of fruit and vegetable freshness [38]. Figure 5B shows the change in weight loss of WBS subjected to different pre-cooling treatments during storage. The weight loss gradually increased in all groups with the extension of storage time. Compared to the CK group, the weight loss of FIPC group was slightly higher at the same time during storage (*p* > 0.05), but the other groups were lower (*p* < 0.05), particularly the CSAEW and CWPC groups. Suathong et al. [39] suggested that respiration was the primary cause of quality loss in vegetables. The FIPC treatment may destroy the cellular structure of WBS during pre-cooling, thereby increasing the respiration intensity, accelerating cellular metabolism and nutrient depletion, finally resulting in the greatest weight loss. Water-mediated pre-cooling treatments might reduce the water loss and prevent cellular damage, which may be the main reason for lower weight loss of WBS in the CSAEW and CWPC groups. It was also related to its lower respiration rates. Zainal et al. [40] found that the water-cooling treatment reduced the weight loss in rock melon during storage. The weight loss observed in the RPC, SWPC, and VPC groups was moderate, while the VPC group showed relatively more weight loss. This may be attributed to the water spraying treatment applied to the surface of WBS in advance during the VPC treatment, which likely mitigated the water loss. Zhang et al. [41] discovered that combining VPC with water spraying effectively reduced the weight loss in cooked carrots and broccoli. Tian et al. [42] also demonstrated that water spraying during VPC reduced the weight loss in broccoli.

### 3.4. Relevant Indices of Lignification in WBS

**Figure 6:**
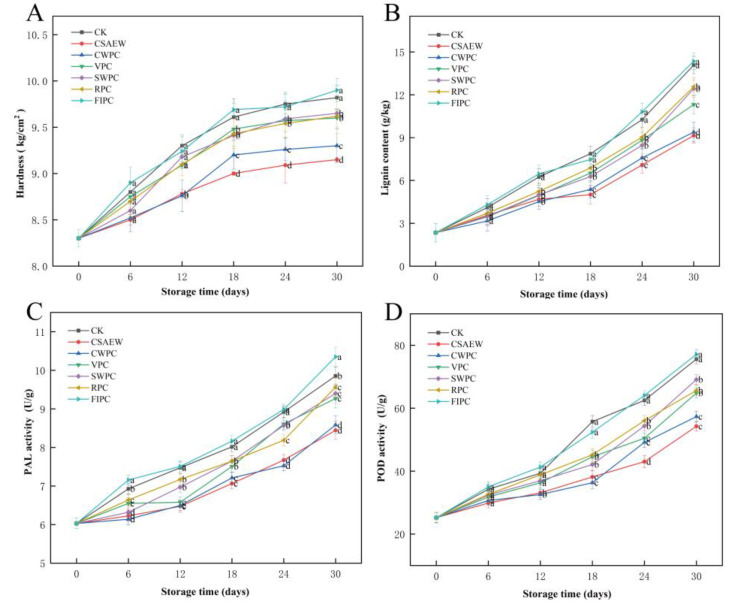
The change in (**A**) hardness, (**B**) lignin content, (**C**) PAL activity, and (**D**) POD activity of WBS subjected to different pre-cooling treatments during storage. The different abbreviations CK, CSAEW, CWPC, VPC, SWPC, RPC, FIPC in the figure represent control group, cold slightly acidic electrolytic water pre-cooling, cold water pre-cooling, vacuum pre-cooling, strong wind pre-cooling, refrigerator pre-cooling, and fluid ice pre-cooling, respectively. Same superscript lowercase letters in a column indicate no significant differences (*p* > 0.05).

WBS is a root vegetable, and its hardness serves as a critical indicator of its quality. The change in the hardness of the WBS is primarily attributed to its lignification and the natural thickening of cell walls [43]. Figure 6A shows the change in hardness of the WBS subjected to different pre-cooling treatments during storage. The initial hardness of the WBS was 8.3 kg/cm^2^, and the hardness of the WBS in all groups gradually increased throughout the storage period. This increase can be attributed to the accumulation of lignin, leading to the lignification of plants [27]. In the early stage of storage, the hardness of the WBS increased relatively rapidly in all groups, and it increased relatively slowly after 18 days of storage. This may be caused by water loss. The water loss altered the cellular structure and metabolic balance of vegetables, leading to wilting and softening [44]. Lignin accumulation leads to hardening of WBS, whereas water loss results in softening. In the early stages of storage, the water loss is modest, with lignin accumulation being predominant. However, the water loss became more significant in the later stage of storage, thereby delaying further increases in hardness. Compared to the CK group, FIPC exacerbated the increase in the hardness of the WBS. This may be due to the low temperature of fluid ice, causing frostbite to WBS during pre-cooling treatment, thereby destroying tissue cells. Frostbite induced the dehydration of plant cells and formation of ice crystals within thin-walled cells. This process not only compromised the integrity of cell membranes, leading to cell death, but also oxidized cellular contents through enzymatic activity, thus affecting physiological functions [45]. The five remaining pre-cooling treatments mitigated the increase in hardness of the WBS to varying degrees, significantly different from the CK group (*p* < 0.05). This suggested that effective pre-cooling treatment was helpful to maintain the hardness of vegetables [46]. Among all the groups, CSAEW pre-cooling treatment resulted in the least increase in WBS hardness, followed by CWPC, SWPC, VPC, and RPC treatments. Following 30 days in storage, the hardness of WBS in the CSAEW group was 9.15 kg/cm^2^, which represented only a 10.24% increase from the initial value, whereas the FIPC group exhibited a 19.28% increase.

The accumulation of lignin, an aromatic polymer macromolecule present in plant cell walls, is the main cause of lignification in WBS [47]. Figure 6B shows the change in the lignin content of WBS subjected to different pre-cooling treatments during storage. The initial lignin content of WBS was 2.35 g/kg, and the lignin content of all groups of WBS grew steadily throughout storage. Compared to the CK group, the FIPC group exhibited higher lignin accumulation in WBS, while other groups showed lower lignin accumulation, significantly different from the CK group (*p* < 0.05). Among all groups, the lignin content of WBS in the CSAEW and CWPC groups was always at a relatively low level during storage. Lignin accumulation is driven by the promotion of lignin-related synthase activity. This may be attributed to the excessively low temperature of fluid ice treatment, which caused frostbite in WBS, injuring the cellular tissues. Consequently, this resulted in increased lignin-related synthase activity and exacerbated lignin accumulation [48,49]. In contrast, the other groups did not injure the tissues of the WBS and exhibited lower respiration rates. The changes in the lignin content of the WBS during storage were consistent with the changes in hardness, indicating a positive correlation between them. The similar result was also demonstrated by Dong et al. [50].

PAL and POD are crucial enzymes in lignin synthesis. PAL acts as a rate-limiting enzyme, catalyzing the deamination of phenylalanine to cinnamic acid, which is a critical step in lignin synthesis [51]. POD promotes the formation of lignin through the polymerization of monolignols [25]. Figure 6C,D show the change in PAL activity and POD activity of WBS subjected to different pre-cooling treatments during storage. The PAL and POD activities of the WBS in all groups increased steadily during storage, aligning with the changes in lignin content. Yang et al. [52] observed that the PAL and POD enzyme activities in the WBS continuously increased during storage, which was consistent with the findings of this study. Compared to the CK group, the FIPC group exhibited higher PAL and POD activities, while these were lower in other groups. Among all groups, the CSAEW and CWPC treatments showed the most effective inhibition against PAL and POD activities. This also corresponded to the results of lignin content. Zheng et al. [53] demonstrated that inhibiting PAL and POD enzyme activities can delay the lignification process in bamboo shoots.

### 3.5. Nutrients and Oxidation in WBS

**Figure 7:**
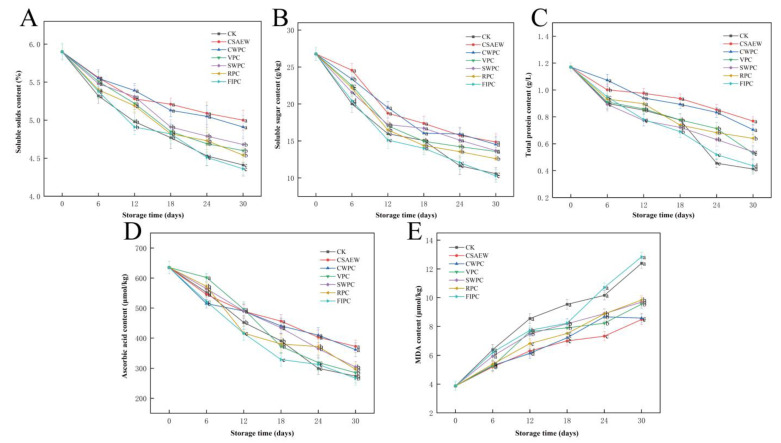
The change in (**A**) soluble solids content, (**B**) soluble sugar content, (**C**) total protein content, (**D**) ascorbic acid content, and (**E**) MDA content of WBS subjected to different pre-cooling treatments during storage. The different abbreviations CK, CSAEW, CWPC, VPC, SWPC, RPC, FIPC in the figure represent control group, cold slightly acidic electrolytic water pre-cooling, cold water pre-cooling, vacuum pre-cooling, strong wind pre-cooling, refrigerator pre-cooling, and fluid ice pre-cooling, respectively. Same superscript lowercase letters in a column indicate no significant differences (*p* > 0.05).

Soluble solids are indicative of the quality of vegetables, encompassing water-soluble sugars, acids, proteins, and other substances that serve as substrates for their respiration [54]. Figure 7A shows the change in soluble solids content of WBS subjected to different pre-cooling treatments during storage. The soluble solids content in all groups decreased with increased storage time. The phenomenon may be explained by the water that was lost from WBS during the last stages of storage, which resulted in a higher concentration of soluble solids and a less varied appearance. The soluble solids content in the FIPC group showed no significant difference from that in the CK group (*p* > 0.05). The other five groups effectively slowed the decline of soluble solids in WBS. The soluble solids content in the CSAEW and CWPC groups was at a relatively high level during storage (significantly different from other groups, *p* < 0.05). After 30 days of storage, the CSAEW and CWPC groups had the highest soluble solids content at 5% and 4.91%, respectively, representing reductions of only 15.25% and 16.78% from their initial values. In contrast, the CK and FIPC groups showed reductions of 25.2% and 26.1%, respectively.

Soluble sugar and total protein are critical nutrients in WBS, serving as essential indicators for assessing the quality and nutritional value of vegetables. They participate in various metabolic processes within these foods. Figure 7B,C show the change in soluble sugar content and total protein content of WBS subjected to different pre-cooling treatments during storage. The soluble sugar and total protein content of WBS in all groups exhibited a decreasing trend with extended storage time. This was mainly due to the depletion of nutrients through respiration during storage. The soluble sugar and total protein content in the FIPC group did not differ significantly from those in the CK group (*p* > 0.05). The other five pre-cooling treatments effectively slowed the decline in soluble sugar and total protein content in WBS. Following 30 days in storage, the CSAEW group exhibited the highest soluble sugar and total protein content, measuring 14.86 g/kg and 0.77 g/L, respectively. The FIPC group had the lowest soluble sugar content (10.28 g/kg), and the CK group had the lowest total protein content (0.41 g/L).

Ascorbic acid is an important primary metabolite for plants that has strong antioxidant effects and can improve plant resilience [55]. Its high antioxidant activity can also inhibit the browning response in plants. Figure 7D shows the change in the ascorbic acid content of WBS subjected to different pre-cooling treatments during storage. As the period of storage increased, the ascorbic acid content of WBS in all groups exhibited a decreasing trend. The browning of micro-processed WBS was observed mainly at the dehulled part and at the bottom, which may be due to the oxidation of ascorbic acid. The mechanical damage caused by post-harvest shelling and cutting may accelerate the oxidation of ascorbic acid. Following 30 days in storage, the CSAEW and CWPC groups maintained high ascorbic acid levels of 372.21 μmol/kg and 361.19 μmol/kg, respectively, significantly different from other groups, *p* < 0.05. The other groups, in descending order, were SWPC (303.97 μmol/kg), RPC (296.56 μmol/kg), VPC (285.36 μmol/kg), CK (273.7 μmol/kg), and FIPC (266.75 μmol/kg). This could be attributed to the relatively low respiration and water loss in the CSAEW and CWPC groups, which favored the mitigation of ascorbic acid oxidation. This was consistent with the appearance changes of WBS during storage. Chen et al. [56] found that acidic electrolyzed water can inhibit the reduction in soluble solids, soluble sugar, and ascorbic acid content of longan fruit.

MDA, a terminal product of lipid peroxidation, serves as an indicator of the integrity of cell membranes in vegetables [57]. Figure 7E shows the change in the MDA content of WBS subjected to different pre-cooling treatments during storage. The MDA content of WBS in all groups exhibited an increasing trend with extended storage time. After 18 days of storage, the MDA level of WBS in the FIPC group exceeded that of WBS in the CK group. It was likely due to the frostbite in the WBS caused by the low temperature of the FIPC treatment, which induced oxidative stress and subsequent cell membrane damage. Reactive oxygen species (ROS), byproducts of cellular metabolism in vegetables, are influenced by environmental factors and regulated by non-enzymatic reactions such as those involving ascorbic acid. The accumulation of excess ROS can damage cell membranes [58]. Consequently, MDA content increased significantly during the late storage period, correlating with prior observations of respiration rate, soluble solids, and other markers. The MDA content in other groups was significantly lower than that of the CK group, indicating that CSAEW, CWPC, VPC, SWPC, and RPC effectively inhibited MDA accumulation. This suggests that these pre-cooling treatments preserved cell membrane integrity and prevented lipid peroxidation. Li et al. [29] demonstrated that low temperatures inhibited MDA accumulation in apricots, thereby protecting cell membrane function. Among all groups, the CSAEW group maintained a comparatively low MDA level during storage and its MDA content was 8.48 μmol/kg on the 30th day, which was 31.56% lower than that of CK group (*p* < 0.05). This indicated that the CSAEW treatment can diminish excessive ROS accumulation and enhance the antioxidant capacity of WBS. This was further evidenced by the highest ascorbic acid content in CSAEW group.

### 3.6. Total Bacterial Colony Count

**Figure 8:**
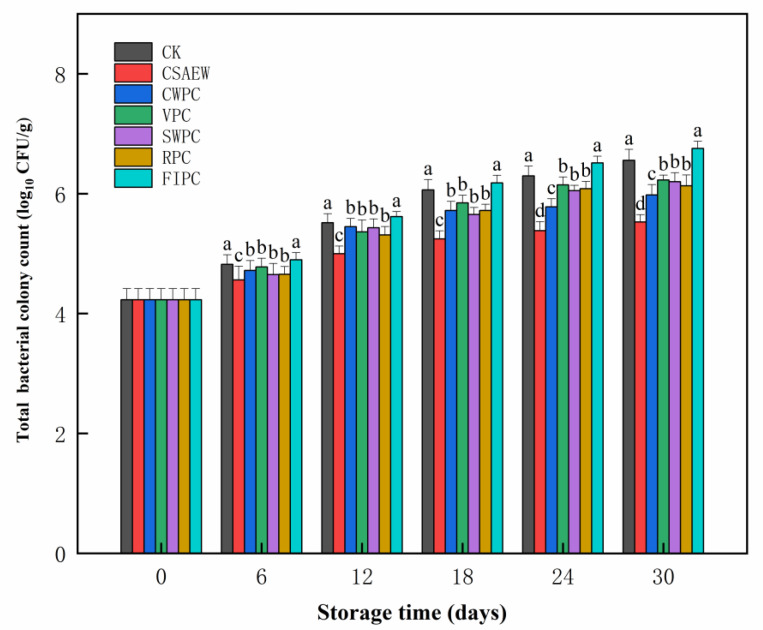
The change in total bacterial colony count of WBS subjected to different pre-cooling treatments during storage. The different abbreviations CK, CSAEW, CWPC, VPC, SWPC, RPC, FIPC in the figure represent control group, cold slightly acidic electrolytic water pre-cooling, cold water pre-cooling, vacuum pre-cooling, strong wind pre-cooling, refrigerator pre-cooling, and fluid ice pre-cooling, respectively. Same superscript lowercase letters in a column indicate no significant differences (*p* > 0.05).

The total bacterial colony count serves as a critical microbiological indicator of freshness in vegetables [59]. Figure 8 shows the change in the total bacterial colony count of WBS subjected to different pre-cooling treatments during storage. Over the extended storage period, the total bacterial colony count of WBS in all groups exhibited an increasing trend. Compared to the CK group, the FIPC group showed no significant difference (*p* > 0.05), whereas other groups exhibited varying degrees of inhibitory effects on colony growth. The phenomenon could be explained by pre-cooling after harvest, which quickly removed field heat and produced an environment that was less favorable for microbial growth and reproduction. It was evident that the total bacterial colony count in the CSAEW group was significantly lower than that of other groups during the same storage period (*p* < 0.05). This was attributed to the antibacterial and bactericidal effects of SAEW. Hypochlorous acid was a primary functional component of SAEW, which can disrupt bacterial cell membranes and serve as a bactericide. SAEW has been demonstrated to effectively inhibit the growth and proliferation of microorganisms in vegetables during storage. Li et al. [22] found that electrolyzed water effectively reduced the microorganisms in fresh-cut eggplant during storage. Ding et al. [60] observed that SAEW effectively reduced the total bacterial colony count, yeast, and mold in strawberries and cherry tomatoes. Zhang et al. [61] reported that SAEW reduced the total bacterial colony count in bean sprouts. By the 18th day of storage, the cumulative colony counts in the CK and FIPC groups exceeded 6 log_10_ CFU/g, indicating that they were inedible [62]. By the 24th day of storage, the overall colony counts in the SWPC, VPC, and RPC groups all exceeded 6 log_10_ CFU/g. By the 30th day of storage, the cumulative colony counts in the CSAEW and CWPC groups were 5.53 log_10_ CFU/g and 5.98 log_10_ CFU/g, respectively, indicating that they were still edible. According to this microbiological indicator, the shelf life of WBS with different pre-cooling treatments can be divided into three levels: CSAEW and CWPC groups were the first level (30), VPC, SWPC, and RPC groups were the second level (24 d), and CK and FIPC groups were the last level (18 d).

## 4. Conclusions

The effects of various pre-cooling treatments (CSAEW, CWPC, VPC, SWPC, RPC, and FIPC) on the quality of refrigerated WBS were compared. The results showed that the FIPC treatment was unsuitable for maintaining the storage quality of WBS, while the other five pre-cooling treatments could extend the shelf life of WBS to some extent. These pre-cooling treatments can reduce the respiration rate of WBS, slow down its weight loss and lignification process, and maintain its relatively high levels of nutrient content and antioxidant activity. The protective effect of CSAEW treatment on the storage quality of WBS was relatively the best. The shelf life of WBS with different pre-cooling treatments can be divided into three levels: CSAEW and CWPC groups were the first level (30 d), VPC, SWPC, and RPC groups were the second level (24 d), and CK and FIPC groups were the last level (18 d). CSAEW outperformed CWPC in terms of bactericidal action and microbiological control for WBS during storage. The worse quality of WBS with FIPC treatment might be due to tissue injury for the low temperature of fluid ice. The results indicated that the CSAEW pre-cooling treatment exhibits good prospects in pre-cooling vegetables. It provides a new option for pre-cooling vegetables.

## Data Availability

The original contributions presented in the study are included in the article: further inquiries can be directed to the corresponding author.
